# *Coxiella burnetii* Induces Inflammatory Interferon-Like Signature in Plasmacytoid Dendritic Cells: A New Feature of Immune Response in Q Fever

**DOI:** 10.3389/fcimb.2016.00070

**Published:** 2016-06-27

**Authors:** Mignane B. Ka, Soraya Mezouar, Amira Ben Amara, Didier Raoult, Eric Ghigo, Daniel Olive, Jean-Louis Mege

**Affiliations:** ^1^Unité de Recherche sur les Maladies Infectieuses Tropicales et Emergentes, UMR 63, Centre National de la Recherche Scientifique 7278, INSERM U1095, IRD 198, Aix-Marseille UniversitéMarseille, France; ^2^INSERM UMR 1068, Centre de Recherche en Cancérologie de MarseilleMarseille, France

**Keywords:** plasmacytoid dendritic cells, *Coxiella burnetii*, Q fever, interferon, infection

## Abstract

Plasmacytoid dendritic cells (pDCs) play a major role in antiviral immunity *via* the production of type I interferons (IFNs). There is some evidence that pDCs interact with bacteria but it is not yet clear whether they are protective or contribute to bacterial pathogenicity. We wished to investigate whether *Coxiella burnetii*, the agent of Q fever, interacts with pDCs. The stimulation of pDCs with *C. burnetii* increased the expression of activation and migratory markers (CD86 and CCR7) as determined by flow cytometry and modulated gene expression program as revealed by a microarray approach. Indeed, genes encoding for pro-inflammatory cytokines, chemokines, and type I INF were up-regulated. The up-regulation of type I IFN was correlated with an increase in IFN-α release by *C. burnetii*-stimulated pDCs. We also investigated pDCs in patients with Q fever endocarditis. Using flow cytometry and a specific gating strategy, we found that the number of circulating pDCs was significantly lower in patients with Q fever endocarditis as compared to healthy donors. In addition, the remaining circulating pDCs expressed activation and migratory markers. As a whole, our study identified non-previously reported activation of pDCs by *C. burnetii* and their modulation during Q fever.

## Introduction

Plasmacytoid dendritic cells (pDCs) are a subset of DCs present in the blood and lymphoid organs and are well-documented as the major type I interferon (IFN)-producing cells (Cella et al., 1999; Colonna et al., 2002). Their resting phenotype is characterized by the expression of chemokine receptors such as CCR5, CXCR4, and microbe sensors including C-type lectins, TLR7, and TLR9. Once activated, they overexpress two specific markers, CD86 (activation) and CCR7 (migratory marker), and produce high levels of type I IFN and inflammatory cytokines (Siegal et al., 1999; Facchetti et al., 2003; Liu, 2005; Martinelli et al., 2007; Gilliet et al., 2008). Plasmacytoid dendritic cells specialize in innate antiviral immunity and are also involved in adaptive immunity (Liu, 2005; Fiorentini et al., 2008). Type I IFNs are produced transiently by pDCs in acute viral infections and have a limited amplitude (Swiecki and Colonna, 2015). Although produced by pDCs in chronic viral infections such as those due to Human Immunodeficiency Virus (HIV; Bowie and Unterholzner, 2008; Sachdeva et al., 2008), hepatitis C virus (Mengshol et al., 2009), and hepatitis B virus (Xie et al., 2009), the role of type I IFNs is more complex. Indeed, pDCs may contribute to chronicity *via* the dysregulation of type I INF production (Swiecki and Colonna, 2015).

In contrast to viral infections, the role of pDCs in the defense against bacteria is poorly understood. There are few examples of pDC maturation in response to bacteria *in vitro. Streptococcus pyogenes* (Veckman and Julkunen, 2008) and *Mycobacterium tuberculosis* (Lozza et al., 2014) increase the maturation of human pDCs, leading to the activation of naive CD4^+^ T cells and Th1 polarization. The ability of pDCs to produce type I IFNs in response to bacterial infections depends on the bacterial strain. While *Borrelia burgdorferi* and *Staphylococcus aureus* induce the production of type I IFNs by pDCs, *S. pyogenes* and *Legionella pneumophila* do not (Veckman and Julkunen, 2008; Eberle et al., 2009; Bekeredjian-Ding et al., 2014). In addition, pDCs may be protective against bacterial infections. Hence, the depletion of pDCs in a murine model of *Chlamydia pneumoniae* infection results in severe and prolonged chronic inflammation (Crother et al., 2012).

The presence of *Coxiella burnetii*, the bacterium responsible for Q fever, within pDCs was recently reported in the lymph nodes of patients with lymphomas (Melenotte et al., 2016). This surprising observation led us to investigate the role of pDCs in Q fever. Q fever is an infectious disease characterized by a primary-infection which may become chronic in patients with an altered immune response. The chronic expression of the disease is dominated by endocarditis or vascular infection (Raoult et al., 2005). Despite numerous studies, the mechanisms of Q fever endocarditis and vascular infection remain partially understood. We, and other teams, have reported the modulation of circulating leukocytes, including monocyte and T cell subsets (Ka et al., 2014, 2015), a non-protective inflammatory response (Honstettre et al., 2003; Schoffelen et al., 2013) and the prominent role of IL-10 in bacterial persistence and defective microbicidal activity (Amara et al., 2012). Although, it is largely admitted that monocytes and macrophages are the targets of *C. burnetii*, there is evidence that other cell types, including DCs, may host the microorganisms (Shannon et al., 2005). We recently showed that *C. burnetii* activates human monocyte-derived dendritic cells (moDCs), inducing a transcriptional inflammatory program in which the type I IFN pathway is impaired (Gorvel et al., 2014). However, the interaction of *C. burnetii* with other DC populations has not yet been reported.

In this report, we show that *C. burnetii* induces a migratory phenotype and a specific inflammatory signature in pDCs. *Coxiella burnetii* also stimulates the release of type I IFNs. In addition, the number of circulating pDCs was lower in patients with Q fever endocarditis. Taken as a whole, the present study shows that pDCs are involved in *C. burnetii* infection and identifies a new feature of the immune response in Q fever.

## Materials and methods

### Patients with Q fever endocarditis

The study of 17 patients with Q fever endocarditis (consisting of six women and eleven men aged between 22 and 79 years old) and their controls was conducted with the approval of the Ethics Committee of Aix-Marseille University (Marseille, France) and with the written consent of each participant. The characteristics of the patients were previously described (Ka et al., 2014). Seventeen patients with Q fever endocarditis were included on the basis of the presence of endocarditis, a positive echocardiogram and blood culture, high titers of IgG specific for phase I *C. burnetii* and data scoring (Raoult, 2012). Ten age- and sex-matched individuals were included as healthy controls.

### Bacteria production and preparation

*Coxiella burnetii* (Nine Mile strain, RSA496) was cultured as previously described (Gorvel et al., 2014). The L929 cells were infected for 8 days and were sonicated and centrifuged at 300 × *g* for 10 min. Supernatants were collected and centrifuged at 10,000 × *g* for 10 min. Bacteria were then washed and stored at −80°C. The concentration of organisms was determined by Gimenez staining and bacterial viability was assessed using the LIVE/DEAD BacLight bacterial viability kit (Molecular Probes, Life Technologies).

### pDC isolation and stimulation

Leukopacks were obtained from the Etablissement Français du Sang. Peripheral blood mononuclear cells (PBMCs) were recovered using density gradient centrifugation. The pDCs were isolated using magnetic beads on AutoMacs (Miltenyi Biotech) as previously described (Ka et al., 2014). Briefly, pDCs were isolated by depletion of non-pDCs that were retained in the column, while unlabeled pDCs with high purity (90%) were collected in the flow-through. Plasmacytoid dendritic cells were then suspended in RPMI 1640, supplemented with 20 mM HEPES, 10% fetal calf serum, 2 mM L-glutamine, 100 U penicillin/ml, 50 μg/ml streptomycin (Life Technologies), and 10 ng/ml recombinant IL-3 (R&D Systems), as described previously (Dental et al., 2012) and were stimulated with heat-inactivated (100°C for 30 min) *C. burnetii* organisms (bacterium-to-cell ratio of 50:1), or CpG-A 10 μg/mL (ODN 2216; InvivoGen) for 8 or 24 h.

### Flow cytometry

Flow cytometry was used to study isolated pDCs within PBMCs. First, isolated pDCs were analyzed according to the expression of activation (CD86) and maturation (CCR7) markers. In brief, after stimulation, isolated-pDCs were washed and then incubated with CD86 or CCR7 antibodies or isotypic controls for 20 min. After fixation with 4% paraformaldehyde, the expression of markers was evaluated by flow cytometry. Second, PBMCs from patients with Q fever endocarditis or healthy controls were incubated with specific antibodies or isotypic controls and Aqua-Fluorescent Reactive dye (Aqua-vivid), a viability dye, for 20 min (Supplementary Table 1). PBMCs were then washed and fixed in 4% paraformaldehyde. Flow cytometry was performed using an LSRII-SORP cytometer (Becton Dickinson). After 500,000 events had been completed and debris on the forward/side scatter dot plot and dead cells had been excluded with Aqua-Fluorescent Reactive dye, data were exported and analyzed with FlowJo Software (version 9.2, Tree Star Ashland) (Ka et al., 2014). The proportion of pDCs is expressed as the ratio of living cells expressing the fluorescent marker to the total number of analyzed PBMCs. The phenotypic expression of HLA-DR and PD-1 is expressed as the mean fluorescence intensity (MFI).

### Microarrays

Isolated pDCs were stimulated with heat-inactivated *C. burnetii* for 8 h. RNA was extracted using RNeasy Mini Kits (Qiagen) with a DNase I step to eliminate DNA contaminants, as previously described (Gorvel et al., 2014). The quantity and quality of the RNA was assessed using a Nanodrop spectrophotometer (Thermo Science) and a 2100 Bioanalyzer (Agilent Technologies), respectively. The microarray study was performed using the technology provided by Agilent Technologies, consisting of microarray chips including 45,000 probes (4x44K Whole Human Genome G4112F) and One-Color Microarray-Based Gene Expression Analysis. In brief, 400 ng RNA were labeled with cyanine-3 CTP using a Low RNA Input Fluorescent Amplification kit. Three biological replicates per group were labeled using a QIAamp labeling kit, deposited on microarray slides, and hybridized for 17 h at 65°C. The microarray slides were scanned with a pixel size of 5 μm using a DNA Microarray scanner G2505B. The scanned images were analyzed with Feature Extraction Software 10.5.1. The data processing and analysis were performed using the R software package (version 2.15). The raw data were filtered and normalized using the Agi4x44 PreProcess library. Unsupervised and supervised analyses were performed using hierarchical clustering and a principal component analysis. Genes were considered to be differentially expressed if the False Discovery Rate was below 0.1% and the absolute fold change was above 2.0 (Gorvel et al., 2014).

### Quantitative real time-polymerase chain reaction (qRT-PCR)

Specific genes of stimulated pDCs that were found to be highly modulated by microarray were selected and validated by qRT-PCR, as previously described (Ben Amara et al., 2013). Briefly, first-strand cDNA was obtained using oligo(dT) primers and a reverse transcription of 150 ng RNA using a Moloney murine leukemia virus-reverse transcriptase kit (Life Technologies), a ABI7900 Fast Real-Time PCR System, and a SYBR Green Fast Master Mix (Roche Diagnostics). The results were normalized to the housekeeping gene β-actin (ACTB) and were expressed as the median of fold change = 2^−ΔΔCt^, where ΔΔCt = (Ct_Target_ − Ct_Actin_)_assay_ − (Ct_Target_ − Ct_Actin_)_control_, as previously described (Ben Amara et al., 2010).

### Immunoassays

Isolated pDCs were stimulated with heat-inactivated *C. burnetii* organisms for 24 h, and the supernatants were centrifuged at 1000 × *g* for 10 min and frozen at −80°C. In parallel, PBMCs from healthy controls and Q fever patients were cultivated for 24 h and supernatants were treated as above. The release of IFN-α, IL-6, TNF-α, and IL-10 was determined using immunoassay kits. The concentration of IL-10 and IFN-α in patients was also analyzed by immunoassay.

### Statistical analysis

Data were analyzed using the Student's *t*-test to analyze *in vitro* data and the Mann–Whitney *U*-test to study Q fever patients. The results are presented as the median or the mean ± *SD* of three independent experiments, and a *p* < 0.05 was considered statistically significant.

## Results

### *Coxiella burnetii* stimulates human pDCs

**Figure 1:**
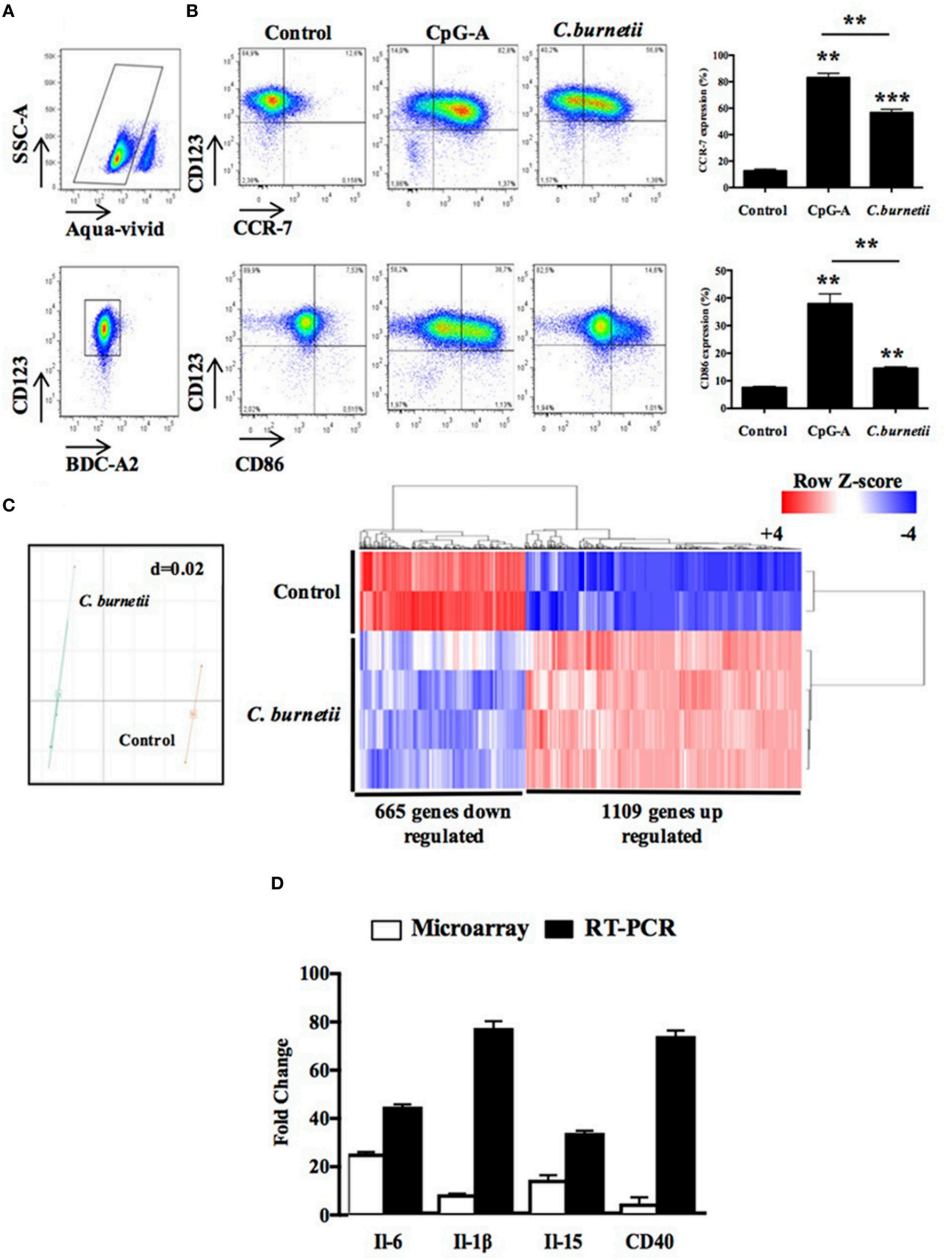
***Coxiella burnetii* modulates an inflammatory response of pDCs. (A)** Flow cytometer graphs showing the gating strategy to study pDCs. **(B)** CCR7 and CD86 expression of isolated pDCs stimulated (*C. burnetii*) or not (control) with heat-inactivated *C. burnetii* or CpG-A (10 μg/ml) during 24 h were visualized **(left panel)** and quantified **(right panel)** by flow cytometer. **(C)** A microarray approach allowed to show the degree of the variation of gene expression **(C, left panel)**, the transcriptional signature **(C, right panel)** and the modulated genes of pDCs stimulated (*C. burnetii*) or not (control) with heat-inactivated *C. burnetii* during 8 h. The microarray was conducted with three biological replicates. **(D)** Representative graph evaluating the fold change of IL-6, IL-1b, IL-15, and CD40 genes using the microarray or the qRT-PCR approach were effected and statistically analyzed on the basis of the *Ct*-values. The results are the means ± SD of three independent experiments. ^**^p < 0.01 and ^***^p < 0.001.

We wanted to investigate whether *C. burnetii* interacts with human pDCs. Living pDCs were isolated from the PBMCs of healthy donors and the expression of BDCA2 and CD123, two specific markers of pDCs, was determined by flow cytometry (Figure 1A). After 24 h of stimulation by *C. burnetii*, the expression of the migratory marker CCR7 and the activation marker CD86 significantly increased as compared to unstimulated pDCs although their expression remained lower than that of pDCs activated with CpG-A used as positive control (Figure 1B), demonstrating that pDCs responded to *C. burnetii* stimulation. We completed the investigation of the response of pDCs to *C. burnetii* using an unbiased approach based on transcriptome study with three biological replicates per group. *Coxiella burnetii* induced a gene expression program which was clearly distinct from that of resting pDCs, as shown by principal component analysis (Figure 1C, **left panel**) and hierarchical clustering (Figure 1C, **right panel**). Using multiclass analysis and a fold change of 2.0, we found that 1109 probes were up-regulated and 665 were down-regulated after 8 h of stimulation with *C. burnetii*. The functional annotation enabled keywords enriched in *C. burnetii*-stimulated pDCs to be identified (Table 1). They included an inflammatory response (enrichment score of 17.83), pathogen sensing (enrichment score of 9.93), and a regulation of cell proliferation (enrichment score of 5.63) in up-regulated genes. We selected the genes encoding for IL-6, IL-1β, IL-15, and CD40 because they were highly up-regulated in microarray and we confirmed their over-expression by qRT-PCR (Figure 1D). Taken together, these results show that *C. burnetii* highly stimulates human pDCs.

**Table 1:** **Up-regulated genes of pDCs-stimulated by *C. burnetii***.

**Keywords**	***P*-values**
**INFLAMMATORY RESPONSE (ES: 17.83-463 genes; INFA, INFB1, INFE, IL1A, IL1B, TNF, IL-6, IL28A, IL28B, IL-29, IL12A, IL12B, IL13, IL15, IL8, CCL2, CCL8, CCL8, CXCL2, CXCL9)**	
Cytokine activity	1.2 × 10^−37^
Cytokine cytokine receptor interaction	4.0 × 10^−28^
Immune response	1.9 × 10^−24^
Defense response	7.8 × 10^−21^
Inflammatory response	1.1 × 10^−12^
**PATHOGEN SENSING (ES: 9.93-80 genes; INFA, INFB, INFE, IL1B, IL28A, IL28B, IL-29, IL12A, IL12B, IL-6, IL8, IL13, IL15, TNF, CCL4, CCL10, CCL10, CXCL9, CXCL11)**	
RIG-I-like receptor signaling pathway	5.5 × 10^−18^
Toll-like receptor signaling pathway	2.4 × 10^−17^
Jak-STAT signaling pathway	1.6 × 10^−15^
Cytosolic DNA-sensing pathway	8.4 × 10^−12^
**REGULATION OF CELL PROLIFERATION (ES: 5.63-88 genes; CD38, CD40, TNF, IL1B, IL4, IL6, IL13, IL15, IL12A, IL12B, IL2R)**	
Positive regulation of lymphocytes proliferation	4.9 × 10^−10^
Positive regulation of leukocytes proliferation	6.1 × 10^−10^
Positive regulation of mononuclear cells proliferation	6.1 × 10^−10^
Positive regulation of lymphocytes proliferation	3.7 × 10^−9^
Regulation of lymphocytes proliferation	4.0 × 10^−9^
Regulation of lymphocytes proliferation	4.6 × 10^−9^
Regulation of apoptosis	1.5 × 10^−7^
Regulation of programmed death	1.9 × 10^−7^
Regulation of death cells	2.0 × 10^−7^
Anti-apoptosis	1.5 × 10^−5^
Negative regulation of apoptosis	9.9 × 10^−5^
Negative regulation of programmed death	1.1 × 10^−4^

*Identification of three clusters defined as: Inflammatory response, Pathogens sensing, and regulation of cell proliferation*.

### *Coxiella burnetii* stimulates inflammatory response in pDCs

**Figure 2:**
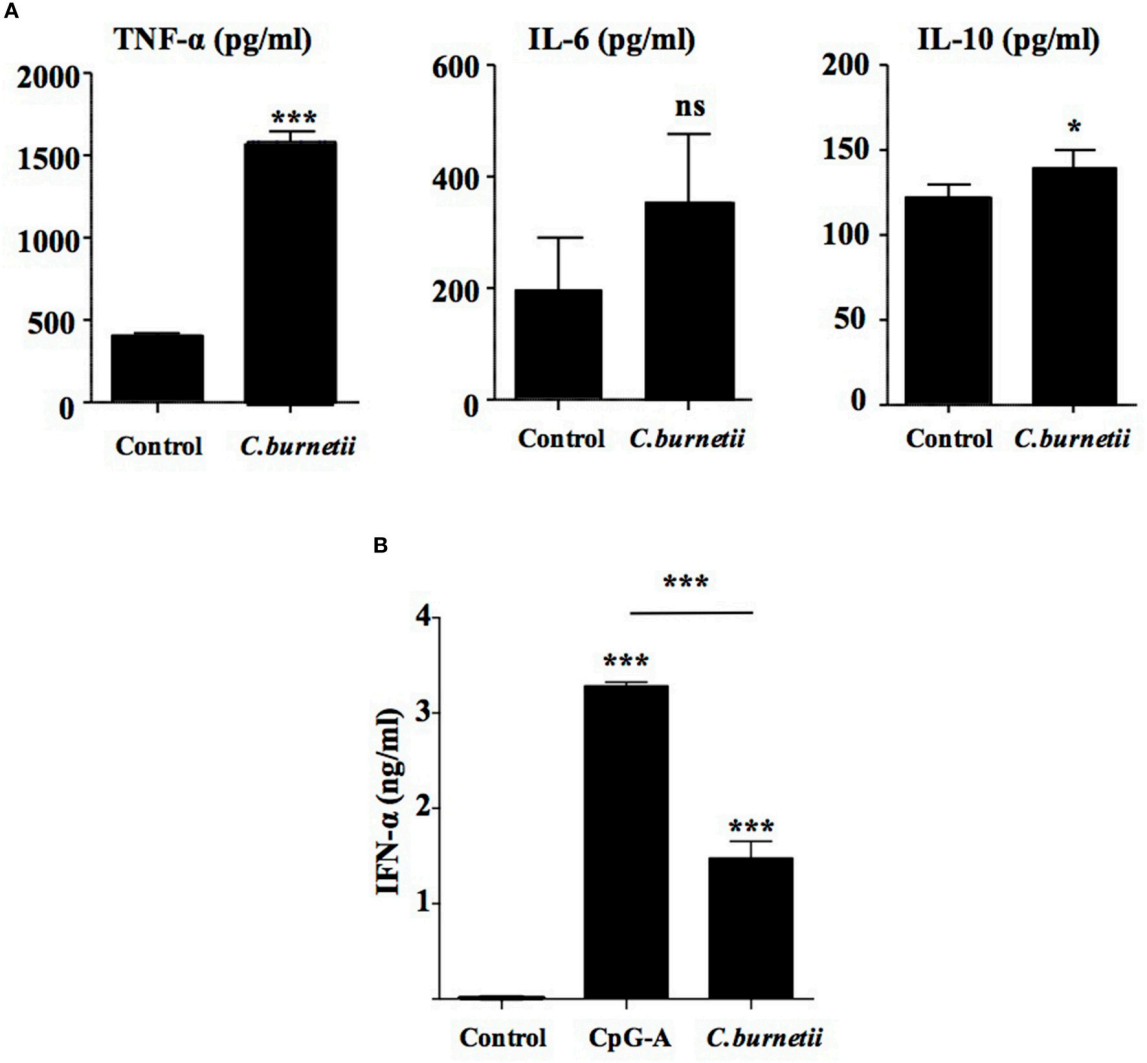
***Coxiella burnetii* induces an interferon-like response by pDCs**. TNF-α **(left panel)**, IL-6 **(middle panel)**, IL-10 **(right panel) (A)** and IFN-a **(B)** were quantified by ELISA immunoassay in supernatants of purified pDCs stimulated with heat-inactivated *C. burnetii* (*C. burnetii*) or not (control) during 24 h. The results are the means ± *SD* of three independent experiments. ^*^p < 0.05 and ^***^p < 0.001.

As inflammatory genes were modulated in *C. burnetii*-stimulated pDCs, we investigated the secretion by pDCs of TNF-α and IL-6, two cytokines considered as inflammatory cytokines. C*oxiella burnetii* induced the release of TNF-α but not that of IL-6. Surprisingly, *C. burnetii* stimulated the release of IL-10, an anti-inflammatory cytokine (Figure 2A), demonstrating subtle changes in the inflammatory response of pDCs to *C. burnetii*. In the microarray signature, two keywords, namely “inflammatory response” and “pathogen sensing” were enriched in *C. burnetii*-stimulated pDCs; they included genes encoding cytokines and chemokines and type I and III IFNs (IFN-α, IFN-β1, and IFN-ε) with a fold change higher than 15 (Table 1). As IFN-α was documented as a cytokine specifically expressed by pDCs, we measured its release in supernatants from pDCs stimulated (or not) with *C. burnetii* or CpG-A, a potent agonist of pDCs. While resting pDCs did not release IFN-α, *C. burnetii*-stimulated pDCs released ~1.5 ng/ml IFN-α (Figure 2B), demonstrating that *C. burnetii* stimulated type I IFN expression at both transcriptional and transductional levels. Taken together, these results showed that *C. burnetii* affected the inflammatory response of pDCs and particularly the IFN-α response.

### Circulating pDCs are affected in patients with Q fever endocarditis

**Figure 3:**
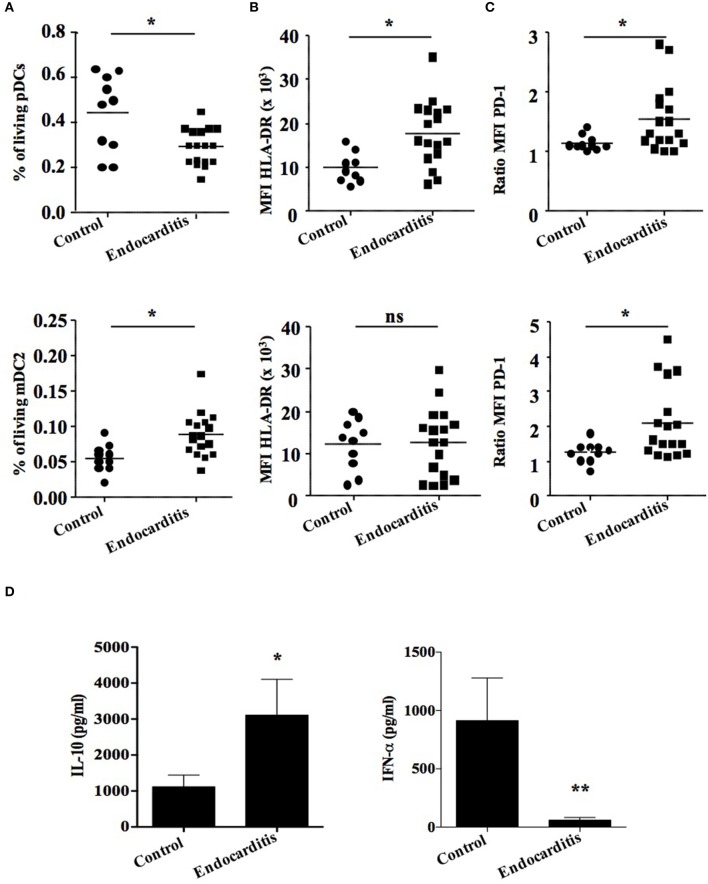
**Plasmacytoid dendritic cells are affected in Q fever patients**. Plasmacytoid dendritic cells from healthy controls (*n* = 10) and patients with Q fever endocarditis (*n* = 17) were analyzed and quantified by flow cytometry and Elisa. **(A)** Graphs showing the percentage of living cells, of **(B)** HLA-DR and **(C)** PD-1 expression of mDC2 and pDCs in the population studied. **(D)** Quantitative graphs of IL-10 **(left panel)** and IFN-a **(right panel)** were evaluated in Q fever endocarditis patients and healthy donors. The non-parametric Mann-Whitney *U*-test was used to compare patient and control groups. ^*^p < 0.05 and ^**^p < 0.01. Horizontal bar, median value.

As pDCs are activated *in vitro* by *C. burnetii*, we wanted to investigate whether they are modulated in patients during Q fever endocarditis. We investigated the pDC population in blood samples from healthy donors (*n* = 10) and Q fever endocarditis patients (*n* = 17). Using flow cytometry and a gating strategy with specific antibodies (Supplementary Table 1), we were able to discriminate between living pDCs and mDC2 within PBMCs (Supplementary Figure 1). The number of pDCs decreased by 30% in patients with Q fever endocarditis as compared to controls (Figure 3A, **up panel**). In contrast, the percentage of mDC2 was higher in patients with Q fever endocarditis (Figure 3A, **down panel**), demonstrating that pDCs were specifically affected by *C. burnetii* infection. We also found that the phenotypic characteristics of pDCs were affected in Q fever endocarditis. Indeed, the expression of activation markers HLA-DR (Figure 3B, **up panel**) and PD-1 (Figure 3C, **up panel**) was increased in pDCs from patients with Q fever endocarditis. It should be noted that only PD-1 increased in mDC2 from the same patients (Figures 3B,C, **down panels**). Finally, we studied the spontaneous release of IFN-α by PBMCs from patients with Q fever endocarditis. We found that the release of IFN-α was markedly depressed in patients compared with healthy controls (Figure 3D, **right panel**), which is likely to be because the number of circulating pDCs was low. The release of IL-10 was increased in patients with Q fever endocarditis (Figure 3D, **left panel**), in accordance with previous results (Capo et al., 1996). Taken together, these results showed that the number of pDCs was depressed in patients with Q fever endocarditis and that the remaining pDCs exhibited specific features of activation.

## Discussion

In this study, we report, for the first time, the ability of *C. burnetii* to activate human pDCs *in vitro*. Indeed, they expressed CCR7 and CD86, migratory and activation markers, respectively. CCR7 plays a role in the migration of pDCs from blood to T cell areas of the lymph nodes and the white pulp of the spleen (Swiecki and Colonna, 2015). Using a high throughput microarray approach, we showed that *C. burnetii* modulated the expression of a large number of genes in which keywords including “inflammatory response,” “pathogen sensing,” and “regulation of cell proliferation” were enriched. We showed that *C. burnetii* stimulated the production of IFN-α associated with the release of TNF-α and IL-10, suggesting a modulation of inflammatory response to the pathogen.

Interestingly, transcriptomic analysis revealed the up-regulation of genes that could be involved in the type I IFN pathway. First, genes enriched in the keyword “pathogen sensing,” such as *TLR7* and signaling molecules such as *MyD88* and *IRF7*, were up-regulated in *C. burnetii*-stimulated pDCs. The overexpression of *TLR7* and *TLR9* with the *IRF7* gene are necessary for type I IFN production by pDCs, suggesting that this pathway may play a role in pDCs' response to *C. burnetii* (Kawai and Akira, 2010; Trinchieri, 2010). Secondly, the RNA helicase retinoic acid-inducible gene I (RIG-I) was up-regulated in *C. burnetii*-stimulated pDCs. RIG-I is a cytoplasmic receptor known to be responsible for triggering type I IFN secretion (Pandey et al., 2009; Kawai and Akira, 2010). Thirdly, *C. burnetii* up-regulated the expression of the genes encoding the nucleotide-binding oligomerization domain-containing protein (NOD)-1 and NOD2 receptors, which may be involved in the production of type I IFN (Pandey et al., 2009; Watanabe et al., 2010). Indeed, *M. tuberculosis* induces type I IFN production through the activation of NOD2 in macrophages (Pandey et al., 2009) and *Helicobacter pylori* through NOD1 in epithelial cells (Watanabe et al., 2010). In addition, *Neisseria meningitidis, Hemophilus influenza*, and *S. aureus* were found to stimulate the secretion of IFN-α and to increase the expression of CD86 on pDCs (Michea et al., 2013). The production of IFN-α by *C. burnetii*-stimulated pDCs associated with the activation phenotype is distinct from the response to *S. pyogenes* in which IFN-α is not produced (Veckman and Julkunen, 2008) and from the *M. tuberculosis* response in which the activation markers are lacking (Lozza et al., 2014).

As *C. burnetii* induced a specific response in pDCs *in vitro*, we questioned whether circulating pDCs are affected in Q fever. We showed that pDCs were decreased in patients with Q fever endocarditis. We studied patients with Q fever endocarditis and not patients with chronic Q fever since this concept is debated among teams involved in Q fever investigation. In addition, preliminary results showed that patients with primary infection also exhibited decreased amounts of circulating pDCs (data not shown). To our knowledge, this is the first report demonstrating the modulation of the number of circulating pDCs in Q fever. We do not know whether the decrease in circulating pDCs reflects their migration to tissues nor the nature of their role in resistance or susceptibility to *C. burnetii*. The role of pDCs in the defense against bacteria is only supported by a report in which the depletion of pDCs increases the virulence of *C. pneumonia* in mice (Crother et al., 2012). Plasmacytoid dendritic cells are protective in viral infections. The number of circulating pDCs is reduced in patients who are chronically infected with hepatitis B virus, hepatitis C virus and HIV (Goutagny et al., 2004; Ulsenheimer et al., 2005; Meyers et al., 2007). This decrease is correlated with a high viral load (Soumelis et al., 2001; Gurney et al., 2004). Interestingly, patients treated with highly active anti-retroviral therapy are marked by a decreased viral load and an increased number of circulating pDCs, suggesting this cell population is involved in the control of HIV infection (Pacanowski et al., 2001; Keir et al., 2002).

We also showed that circulating pDCs were likely to be activated in Q fever. Indeed, they over-expressed HLA-DR and PD-1. This pattern was not specific since it was also found in mDC2 from patients with Q fever endocarditis. The activated phenotype of pDCs in patients with Q fever may account for the increased traffic of pDCs from blood to peripheral tissues. The increased *in vitro* expression of a migratory marker such as CCR7 in response to *C. burnetii* may account for greater traffic to tissues. We recently showed that pDCs are present in the lymph nodes from Q fever lymphomas and are infected with *C. burnetii* (Melenotte et al., 2016). This suggests that *C. burnetii* infection may encourage the traffic of pDCs to lymph nodes. We also observed that patients with Q fever endocarditis exhibited a decrease in IFN-α production by PBMCs. We hypothesized that the decrease in IFN-α production is related to decrease levels of circulating pDCs. Whether IFN-α is involved in the pathophysiology of Q fever will require additional experiments.

In conclusion, we demonstrated that *C. burnetii* induced a strong inflammatory response in pDCs in which type I IFN was specifically enriched. The present study described a new feature of the immune response in Q fever and also suggested the importance of pDCs in chronic bacterial infections.

## Author contributions

Performed the experiments: MK, SM, and AB. Analyzed the data and wrote the paper: DR, JM, DO, EG, and AB.

### Conflict of interest statement

The authors declare that the research was conducted in the absence of any commercial or financial relationships that could be construed as a potential conflict of interest.

## Supplementary material

Supplementary Figure 1 **Gating strategy to study pDC population (A) Representative graphs showing the gating strategy of pDCs identification by flow cytometry**.

Supplementary Table 1 **List of fluorescent reagents used in this study**. (AF, Alexa fluor; APC, Allophycocyanin; ECD, Phycoerythrin-texas red, FITC, Fluorescein isothiocyanate; PB, Pacific blue; PC, Phycoerythrin; PerCP-Cy5.5, Peridinin chlorophyll protein-cyanin 5.5; and PE, Phycoerythrin).
