# Supplementary figures and images for: Coxiella burnetii Induces Inflammatory Interferon-Like Signature in Plasmacytoid Dendritic Cells: A New Feature of Immune Response in Q Fever
Source: Front Cell Infect Microbiol. 2016 Jun 27;6:70. doi: 10.3389/fcimb.2016.00070 (PMC4921463; doi:10.3389/fcimb.2016.00070)

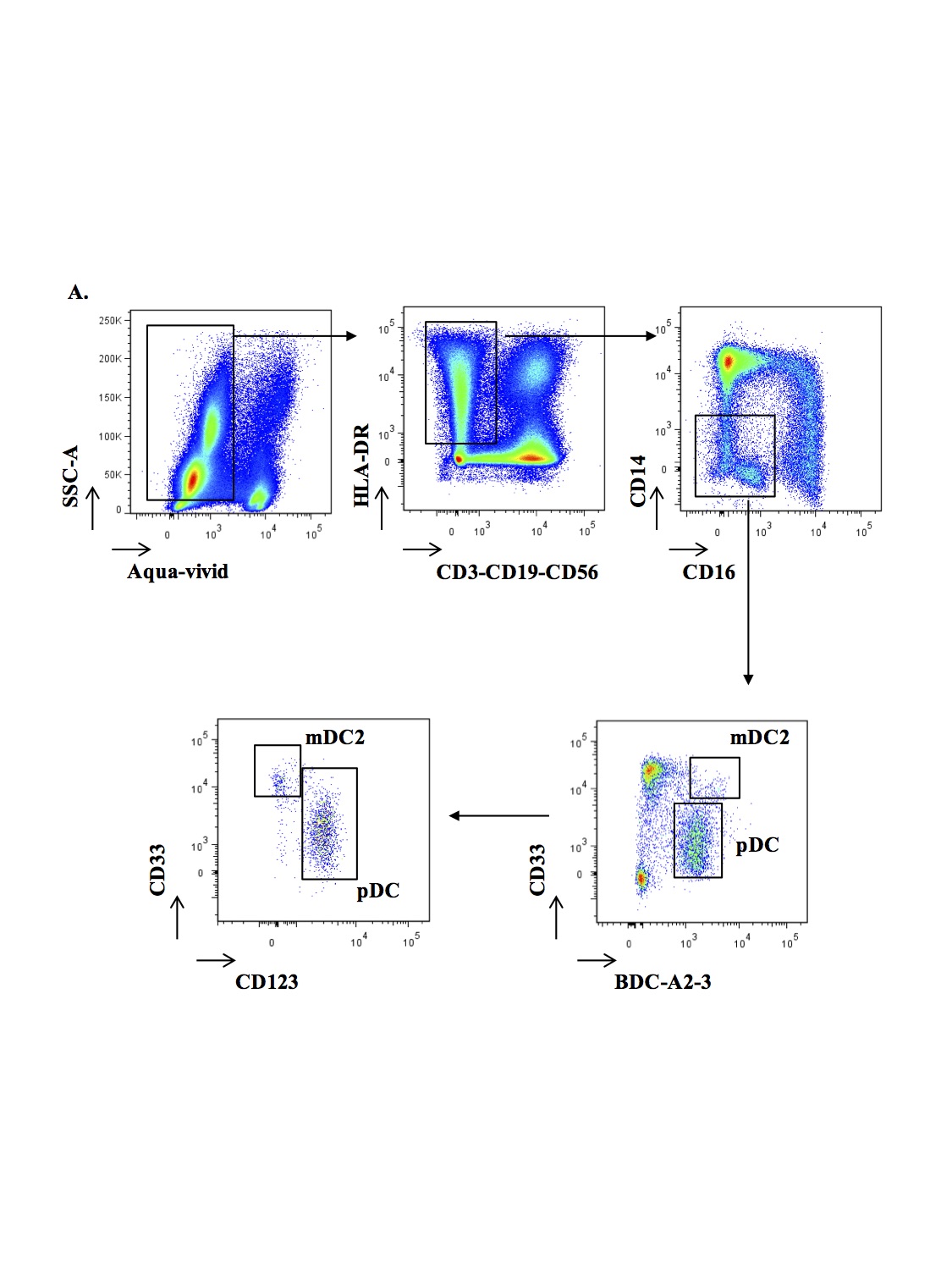

Supplement: Supplementary Figure 1 — Gating strategy to study pDC population (A) Representative graphs showing the gating strategy of pDCs identification by flow cytometry. [file Image1.JPEG]
