# Supplementary material for: Coxiella burnetii Induces Inflammatory Interferon-Like Signature in Plasmacytoid Dendritic Cells: A New Feature of Immune Response in Q Fever
Source: Front Cell Infect Microbiol. 2016 Jun 27;6:70. doi: 10.3389/fcimb.2016.00070 (PMC4921463; doi:10.3389/fcimb.2016.00070)
Supplement: Supplementary Table 1 — List of fluorescent reagents used in this study. (AF, Alexa fluor; APC, Allophycocyanin; ECD, Phycoerythrin-texas red, FITC, Fluorescein isothiocyanate; PB, Pacific blue; PC, Phycoerythrin; PerCP-Cy5.5, Peridinin chlorophyll protein-cyanin 5.5; and PE, Phycoerythrin). [file Table1.docx]

**Supplemental Table 1: List of fluorescent reagents used in this study. (AF**: Alexa fluor**, APC:** Allophycocyanin**, ECD:** Phycoerythrin-texas red**, FITC:** Fluorescein isothiocyanate**, PB:** Pacific blue**, PC:** Phycoerythrin**, PerCP-Cy5.5:** Peridinin chlorophyll protein-cyanin 5.5 **and PE:** Phycoerythrin**).**

| Antibody | mDC2 | pDC | Clone | Specie | Isotype | Fluorochrome | Manufacturer |
| --- | --- | --- | --- | --- | --- | --- | --- |
| CD14 | - | - | MOP9 | Mouse | IgG2b | APC-H7 | Becton Dickinson |
| CD16 | - | - | 3G8 | Mouse | IgG1 | AF700 | Becton Dickinson |
| CD123 | - | + | 7G3 | Mouse | IgG2a | PerCP-Cy5.5 | Becton Dickinson |
| CD86 | / | +  (activated) | HA5.2B7 | Mouse | IgG2b | PC7 | Becton Dickinson |
| CD3 | - | - | OKT3 | Mouse | IgG2a | BV605 | Biolegend |
| CD19 | - | - | HIB19 | Mouse | IgG1 | PE-CF594 | Becton Dickinson |
| CD56 | - | - | B159 | Mouse | IgG1 | PC5 | Becton Dickinson |
| CD33 | + | - | D3HL60.251 | Mouse | IgG1 | PC7 | Beckman Coulter |
| HLA-DR | + | + | Immu-357 | Mouse | IgG1 | ECD | Beckman Coulter |
| CCR7 | / | +  (activated) | 3D12 | Rat | IgG2a | PE | E-Bioscience |
| BDCA-2 | - | + | AC144 | Mouse | IgG1 | FITC | Miltenyi |
| BDCA-3 | + | - | AD5-14H12 | Mouse | IgG1 | FITC | Miltenyi |
| Dead cells |  | | | | | Aqua dye | Life Technologies |
